# Commissioning an in‐room mobile CT for adaptive proton therapy with a compact proton system

**DOI:** 10.1002/acm2.12319

**Published:** 2018-04-06

**Authors:** Jasmine A. Oliver, Omar Zeidan, Sanford L. Meeks, Amish P. Shah, Jason Pukala, Patrick Kelly, Naren R. Ramakrishna, Twyla R. Willoughby

**Affiliations:** ^1^ Orlando Health UF Health Cancer Center Orlando FL USA

**Keywords:** adaptive proton therapy, adaptive re‐planning, hounsfield unit, mobile CT, stopping power ratios

## Abstract

**Purpose:**

To describe the commissioning of AIRO mobile CT system (AIRO) for adaptive proton therapy on a compact double scattering proton therapy system.

**Methods:**

A Gammex phantom was scanned with varying plug patterns, table heights, and mAs on a CT simulator (CT Sim) and on the AIRO. AIRO‐specific CT‐stopping power ratio (SPR) curves were created with a commonly used stoichiometric method using the Gammex phantom. A RANDO anthropomorphic thorax, pelvis, and head phantom, and a CIRS thorax and head phantom were scanned on the CT Sim and AIRO. Clinically realistic treatment plans and nonclinical plans were generated on the CT Sim images and subsequently copied onto the AIRO CT scans for dose recalculation and comparison for various AIRO SPR curves. Gamma analysis was used to evaluate dosimetric deviation between both plans.

**Results:**

AIRO CT values skewed toward solid water when plugs were scanned surrounded by other plugs in phantom. Low‐density materials demonstrated largest differences. Dose calculated on AIRO CT scans with stoichiometric‐based SPR curves produced over‐ranged proton beams when large volumes of low‐density material were in the path of the beam. To create equivalent dose distributions on both data sets, the AIRO SPR curve's low‐density data points were iteratively adjusted to yield better proton beam range agreement based on isodose lines. Comparison of the stoichiometric‐based AIRO SPR curve and the “dose‐adjusted” SPR curve showed slight improvement on gamma analysis between the treatment plan and the AIRO plan for single‐field plans at the 1%, 1 mm level, but did not affect clinical plans indicating that HU number differences between the CT Sim and AIRO did not affect dose calculations for robust clinical beam arrangements.

**Conclusion:**

Based on this study, we believe the AIRO can be used offline for adaptive proton therapy on a compact double scattering proton therapy system.

## 1 INTRODUCTION

Patient weight loss, anatomical changes, and uncertainty in the stopping power of various tissues during proton therapy may cause beam range uncertainty that subsequently compromise tumor coverage and potentially increase dose to healthy tissue.^1^ Adaptive image‐guided proton therapy (IGPT) reduces these dosimetric effects by facilitating the monitoring of patient anatomy during treatment and treatment plan adaptation if necessary.^1^, ^2^ Previously, patients would need to leave the treatment room and undergo an additional CT simulation scan for plan adaptation. However, we recently acquired and commissioned the AIRO Mobile CT System (Mobius Imaging LLC, Ayer, MA, USA) for patient localization and setup (3D IGPT) within the treatment room for our compact proton therapy system.^3^

The AIRO Mobile CT System (AIRO) has historically been used for intraoperative surgeries.^4^ The small footprint (13.81 ft^2^ when in scanning position) of the AIRO allows for CT acquisition in a compact proton therapy vault (Mevion S250, Mevion Medical Systems, Littleton, MA, USA) before or after treatment. The AIRO is a second generation, 32 slice helical CT scanner, capable of acquiring images with 120 kV, 10–250 mA, and field‐of‐view (FOV) up to 51.2 cm. The large FOV allows the scanner to capture the entire patient surface including immobilization devices and the treatment couch.

Most multiroom proton centers do not have in‐room 3D imaging capabilities. Those that do are either limited to one room only with specific adaptations for a CT such as a “track” solution or cone‐beam CT (CBCT). An alternative to mobile helical CT scanners for image‐guided proton therapy is the gantry‐mounted cone‐beam CT (CBCT).^5^, ^6^, ^7^, ^8^, ^9^ Gantry‐mounted CT systems may provide more convenience than mobile scanners. However, CBCT requires gantry‐mounted systems that may not be suitable for compact proton therapy systems. The challenges with reliable proton dose calculation using CBCT images are still unresolved and furthermore, CBCT images require involved offline processing to provide accurate HU and image quality which can be avoided with mobile scanners.^10^

Dose in proton therapy relies on predictably consistent HU (or CT) values and their correlation with accurate stopping powers. The most accurate method of determining a stopping power curve is to scan a phantom with different density plugs that have a known chemical composition so that their relative stopping power can be calculated using stoichimetric methods. The mean CT value of each plug for a particular CT scanner and scan technique is then correlated with that stopping power. The accuracy of the chemical composition, the stopping powers, of each of those elements, and also the noise in the CT value will affect the accuracy of the stopping power.^11^, ^12^ The uncertainties in the stopping power as it relates to the CT values can lead to uncertainties in the calculated range. Along the path of the proton these uncertainties will create small differences in calculated dose. At the end of range where there is a high gradient in dose, the dose difference can be very larger. It is for these reasons that it is customary to include a range uncertainty margin on the proton beam to ensure that the target is covered. In our practice, a margin of 3% of the range is added plus an additional 3 mm to ensure adequate target coverage including uncertainties in the stopping power of the protons. When comparing two different CT scanners for dose comparison, any changes in the CT values and changes in the calculated stopping powers can lead to changes in the dose along the proton path or to a change in the range of the proton therapy.

The purpose of this study was to characterize the AIRO relative stopping power curve in preparation of adaptive proton therapy on a Mevion S250 compact double scattering proton therapy system and to assess the dosimetric implications of adaptive planning with the AIRO imaging system.

## 2 MATERIALS AND METHODS

### 2.A CT number comparison between AIRO and CT Sim

Images of an electron density CT phantom (Gammex RMI 467; Gammex Inc., Middlenton, WI, USA) containing 16 rods of 13 tissue substitute materials were acquired on the CT Sim and on the AIRO (Table 1) with varying plug patterns, table heights, and mA with fixed 120 kV. Thirteen images with various rod placements and table positions were averaged to acquire CT numbers (mean ± standard deviation). For each of the AIRO CT scans, the mobile CT scanner was moved into the proton treatment room and the proton treatment couch was used as the imaging couch top. Analysis of all images was performed in MIM v.6.6.7 software (MIM Software Inc, Cleveland, OH, USA). For each plug, in each scan, the average and standard deviation of the CT numbers for a 1 cm diameter region‐of‐interest (ROI) was acquired. The CT numbers for each plug were compared between the two different scanners. The CT constancy for the AIRO was also tested over several months for the clinically selected protocols.

**Table 1:** Nominal densities and CT numbers of gammex rod materials

Rod materials	Electron density relative to water	Physical density (g/cm^3^)	CT Sim average CT value (HU)	AIRO average CT value (HU)	Relative stopping power (Clinical CT Sim)
LN‐300 Lung	0.28	0.30	297.36	315.32	0.28
LN‐450 Lung	0.40	0.41	450.71	464.75	0.44
Adipose	0.90	0.92	905.69	902.46	0.95
Breast	0.96	0.98	956.58	947.48	0.98
CT solid water	0.99	1.02	996.92	992.79	1.01
Brain	1.05	1.05	1020.03	1004.92	1.03
Liver	1.07	1.11	1070.89	1051.02	1.07
Inner bone	1.09	1.14	1210.49	1209.70	1.13
B200 bone material	1.11	1.16	1221.89	1214.12	1.13
CB2–30% CaCO_3_	1.28	1.34	1439.98	1423.25	1.24
CB2–50% CaCO_3_	1.47	1.56	1799.79	1791.62	1.43
Cortical bone	1.69	1.82	2187.78	2181.50	1.65

**Figure 1:**
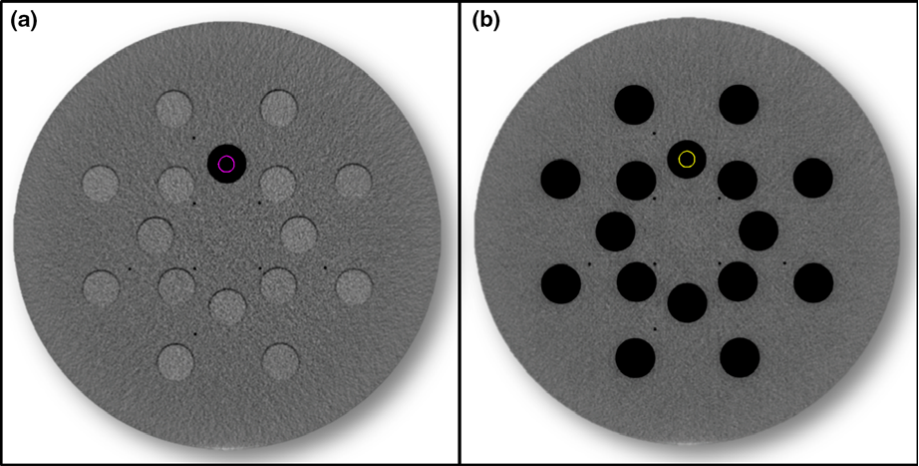
An axial image of the Gammex phantom showing the LN300 low‐density plug with solid water surround (a) and air surround (b).

Images were acquired for the following individual plugs: brain, lung 300, lung 450, cortical bone, adipose, breast, liver, solid water, and true water. The plugs were individually scanned in the Gammex phantom with solid water plugs in all other holes and with no plugs in the other holes to simulate the effect of lung‐like scatter conditions (Fig. 1). This study was performed to evaluate changes in the mean and standard deviations of each plug when scanned alone or with other plugs in place. The mean and standard deviations of the CT numbers were compared between the AIRO and the CT Sim to determine if there were differences in the CT number depending on the scanner used.

The Gammex RMI 467 was scanned as described above with the AIRO in the proton treatment room, as would be used for localization and/or adaptive scanning. To acquire SPRs, averaged CT numbers were entered into a stoichiometric SPR calculation algorithm. The resulting AIRO SPR vs. CT number curve for scans (CT calibration curve) with 120 kV tube‐voltage was entered into our Pinnacle treatment planning software v.16.0 (Philips Medical Systems, Fitchburg, WI, USA).

### 2.B Treatment planning and dosimetric analysis

The last step of commissioning was to confirm the dosimetric equivalence for dose calculated on CT scans from the CT Sim and the AIRO. Treatment planning was done in Pinnacle using a double scatter beam module that had been previously commissioned for treatment planning. All treatment planning was done using the scans from the CT Sim with the clinically used CT Sim SPR curve. CT scans of a RANDO anthropomorphic thorax, pelvis, and head phantom as well as CIRS thorax and head phantoms were used for planning. Heterogeneous, single‐field, nonrobust plans were developed on each phantom in order to test the accuracy of dose for proton beams traversing large areas of heterogeneous media. Additionally, clinically realistic plans were generated to test the accuracy of the adaptive system for use in common clinical scenarios and to evaluate clinical metrics such as dose–volume histogram (DVH) changes between the two CT scans. Phantoms were used to ensure that dosimetric changes were only due to changes in the CT scan and not due to clinical changes in the images. Each phantom was scanned twice with AIRO in the proton treatment room.

Using the dynamic workflow module in the Pinnacle treatment planning system, the proton beams were locked from changes to the original, treatment planning CT scan and the beams were copied to the AIRO CT scans following rigid registration. Dose was calculated on the AIRO CT scans with whichever SPR curve was to be tested. To visually inspect the dosimetric comparison, the isodose lines from the treatment planning scan were converted to contours so they could be displayed on the AIRO CT scan. Dose was calculated on the AIRO CT scan using the following SPR curves: (a) CT Sim SPR curve, (b) AIRO‐specific stoichiometric SPR curve (acquired in 2.A), and (c) dose‐adjusted SPR curve (adjusted after visual inspection of isodose lines and CT numbers).

Initial analysis of the dose calculated with different SPR curves involved evaluation of the new calculated isodose lines overlaid to the contours representing the original isodose lines. This visual inspection illustrates areas where the end of range differed between the two CT scans and showed changes primarily in low‐density material. Changes were made to the SPR curve to improve the agreement along the distal end of the isodose lines on several different plans as illustrated in Fig. 4. While adjusting the SPR curve, the water‐equivalent thickness (WET) to several points along the central axis of the beam were recorded to note changes in WET for different stopping power curves in a heterogeneous phantom. This test was done for a beam representing a PA lung field on the RANDO thorax phantom and was also done for a beam representing a prostate field for the RANDO pelvis phantom.

To further compare the dosimetry between the treatment planning CT calculation and the AIRO CT calculation, gamma analysis of distance‐to‐agreement (DTA) and dose difference (DD) was performed using SNC patient software v.6.6 (Sun Nuclear Corporation, Melbourne, FL, USA).^13^ The dose difference was chosen because it was a measure of the overall difference of the dose between the two CT calculations that should be the same. Because of the sharp fall‐off in the dose at the distal edge of the beam, the distance‐to‐agreement was also a good metric to evaluate the image registration between the two images and would be more sensitive to stopping power differences for the beam at the end of range. Each plan was assessed with the following criterion: 3%, 3 mm; 2%, 2 mm; and 1%, 1 mm. A gamma analysis of 1%, 1 mm was assessed to detect small variations in dose. However, larger criterion of 3%, 3 mm was assessed because clinically we utilize a 3% + 3 mm as our range uncertainty for proton plans. Also, this criterion for gamma analysis is utilized for IMRT QA and gives some clinical reference regarding the acceptability of comparing two different dose planes.^13^, ^14^, ^15^, ^16^ Plans were evaluated at treatment isocenter in the coronal, sagittal, and transverse planes. All three stopping power curves (CT Sim, AIRO, and dose adjusted) were assessed and compared to the CT Sim plans. The dosimetry analysis for all plans was repeated on both AIRO scans.

#### 2.B.1 Nonclinical, heterogeneous plan details

For all phantoms, a treatment plan was developed with a single beam that traversed a heterogeneous portion of tissue. On the RANDO thorax phantom, one beam was placed that traversed both lungs and the heart with the end of range stopping in soft tissue. Another beam was placed from the posteriorly which traversed a very small amount of soft tissue density with the proton beam stopping within lung tissue. For the CIRS phantom, a single beam was placed posteriorly to traverse through both high Z “spine” material and low Z “lung” material. For the RANDO head phantom, a single beam was placed through anteriorly that passed through sinus, soft tissue, and the base of skull. A similar beam was used for the single‐field CIRS phantom plan. The RANDO single‐field pelvis plan was through a single hip. For each of these single‐field plans, the locked beams from the CT Sim plan were copied to the AIRO CT scan for dose recalculation using the three different SPR curves.

#### 2.B.2 Clinically realistic plans

On the RANDO thorax phantom, a spherical planning target volume (PTV) was placed onto the posterior right upper lobe. A three‐field plan was developed with posterior–anterior (PA) field, right posterior oblique (RPO) field, and right anterior oblique (RAO) field. Each field was designed with 3%, 3 mm range uncertainty margin and water‐equivalent modulation margin with appropriate edge processing and smearing margin that would be expected for lung planning at our facility. The PTV was prescribed 60 Gy in 30 fractions. On the RANDO pelvis phantom, a prostate PTV was adapted from a clinical treatment plan containing two fields (right and left lateral). The plan used similar range and modulation margin as well as appropriate edge processing and smearing values. On the RANDO head phantom, a spherical PTV was placed in the medial posterior brain. A three‐field plan with beam angles similar to a beam arrangement, and beam parameters used clinically was used. The CIRS phantom thorax and head phantoms were also planned in a similar manner. In total there were five different clinically reasonable plans developed on five different phantoms. Each of these treatment plans were copied onto two different AIRO scans and the dose recalculated for the new CT scan and SPR curves. In addition to the gamma analysis, clinical metrics of the DVHs were evaluated for dose agreement between critical structures on clinical plans only.

## 3 RESULTS

### 3.A CT number comparisons between AIRO and CT Sim

Table 1 shows average CT numbers for the CT Sim and the AIRO. CT number values for the AIRO were within 2.0% from the CT Sim values for all plugs except for the LN‐300 and LN‐450. The CT numbers for these two plugs were 5.7% and 3.0% higher than the CT Sim values for the same plugs, respectively. It was also noted that the low‐density plugs have a higher CT number in the AIRO and the high‐density plugs have a lower CT number when compared to the CT Sim CT scan. These results are averaged over different table positions and different mA for 13 different scans on both the CT Sim and the AIRO.

**Figure 2:**
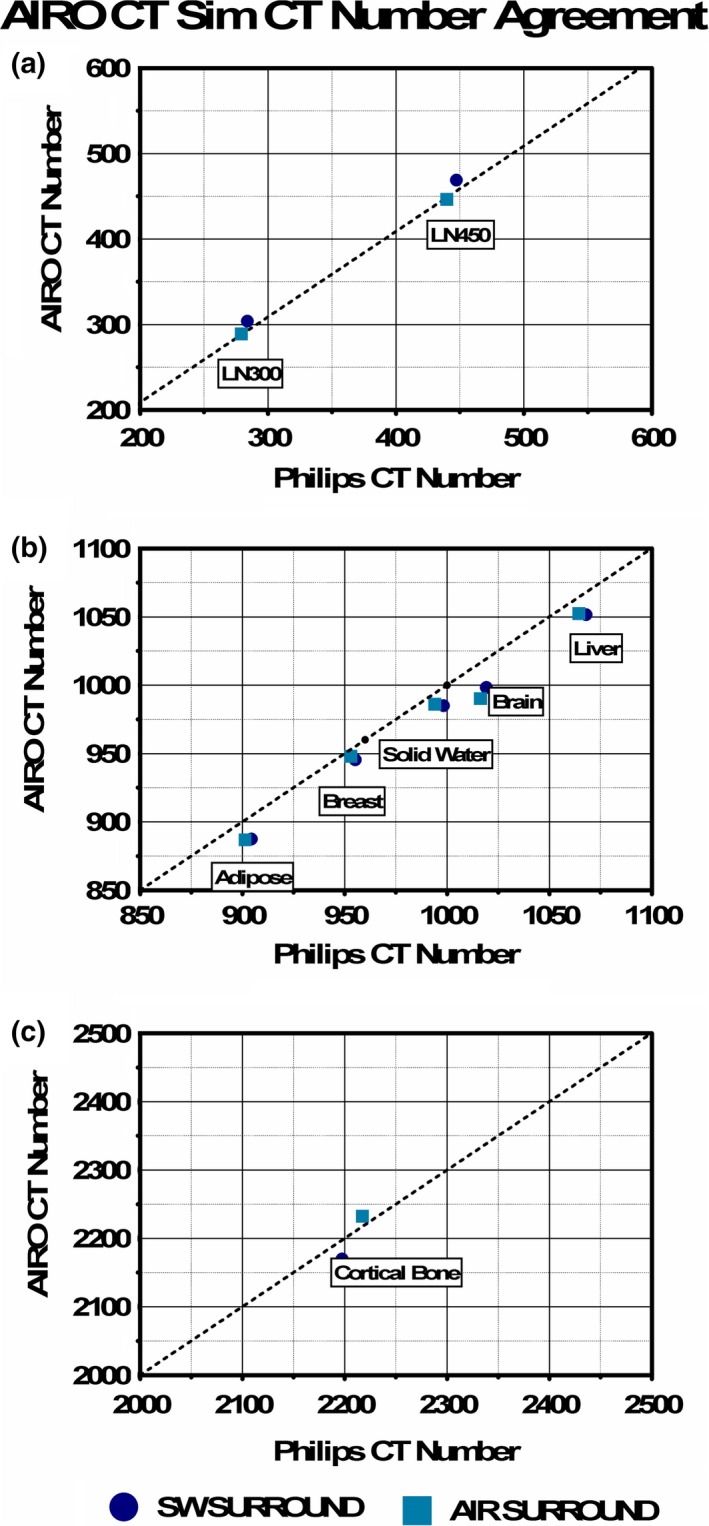
Low‐ and high‐density plug CT number comparison between AIRO and clinical CT Sim scanners. (a) Discrepancies for LN300 and LN450, (b) discrepancies for adipose, breast, solid water, brain, and liver, and (c) discrepancies for cortical bone. Solid water surround is shown in Fig. 1(a). Air surround is shown in Fig. 1(b).

Figure 2 shows CT number comparisons for single plugs on the AIRO and CT Sim. The circles represent the average CT numbers with all surrounding plugs filled with solid water [Fig. 1(a)]. The squares represent the average CT numbers with open plugs labeled Air Surround [Fig. 1(b)]. The difference in the average CT numbers between the two different scan configurations was greater in the AIRO CT scans compared to the CT Sim scans that showed little difference in CT number with scan configurations. This change in CT numbers depending on plug configuration was most notable for the low‐density plugs and also for the high‐density plug. Most of the soft tissue plugs did not change with configuration. Table 2 is a summary of the mean and standard deviation of the CT numbers for each plug with different configurations.

**Table 2:** Mean and standard deviation for individual plugs scanned with air surrounding material vs. solid water surrounding materials on the CT Sim and the AIRO

Rod materials	CT Sim – Air surround		CT Sim – SW surround		AIRO – Air surround		AIRO – SW surround	
	Mean (HU)	SD (HU)	Mean (HU)	SD (HU)	Mean (HU)	SD (HU)	Mean (HU)	SD (HU)
LN‐300 Lung	287.8	23.6	292.6	25.8	289.2	14.2	304.0	18.1
LN‐450 Lung	448.3	25.3	456.1	27.0	446.4	16.9	468.8	17.4
Adipose	901.2	15.4	904.42	23.2	887.0	9.43	887.4	15.4
Breast	952.9	17.0	955.1	23.4	948.1	12.1	945.4	17.0
Solid water	994.0	16.8	998.3	23.0	986.0	11.2	985.1	18.7
Brain	1016.3	13.6	1019.2	17.3	990.3	11.8	998.3	15.2
Liver	1064.3	18.6	1068.0	23.2	1052.4	12.1	1051.5	15.5
Cortical bone	2217.0	22.1	2197.4	32.6	2232.5	15.3	2169.7	26.0

**Figure 3:**
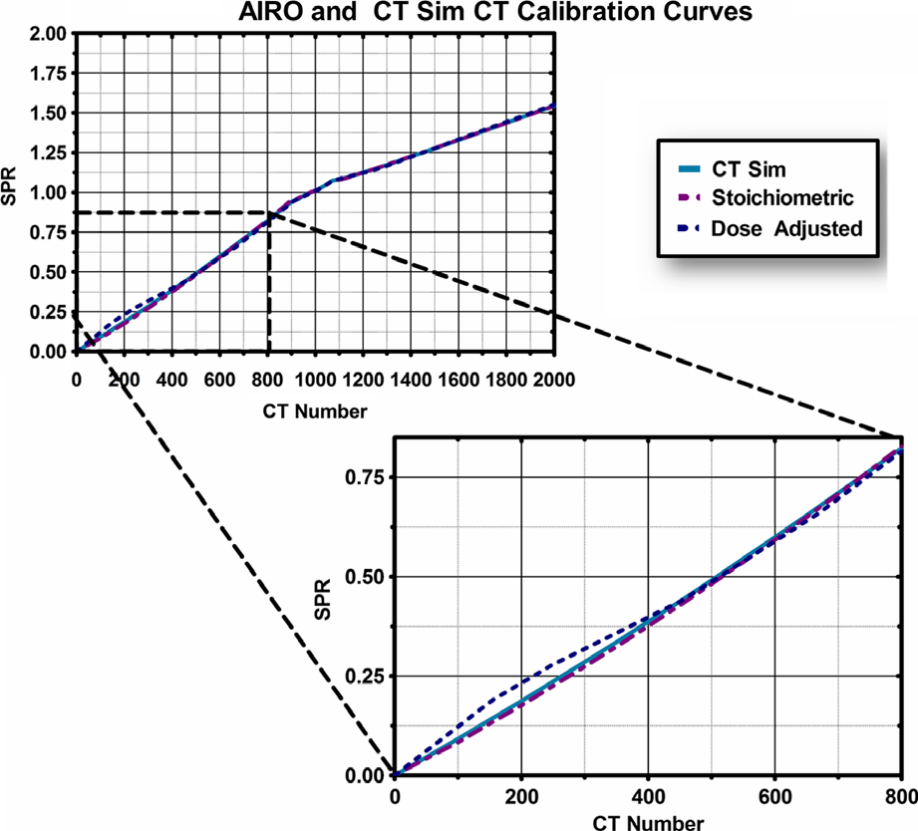
Calculated SPR‐to‐CT calibration curves for AIRO and clinical CT Sim. The CT Sim, AIRO‐specific stoichiometrically developed curve, and dose‐adjusted AIRO CT calibration curve are plotted.

The final CT‐SPR curves for the CT Sim and the AIRO are plotted in Fig. 3. These curves appear similar although there are visible differences in the low CT number region (less than 800) and high CT number region (above 1400).

### 3.B Treatment planning and dosimetric analysis

#### 3.B.1 Dose‐adjusted SPR curve

**Figure 4:**
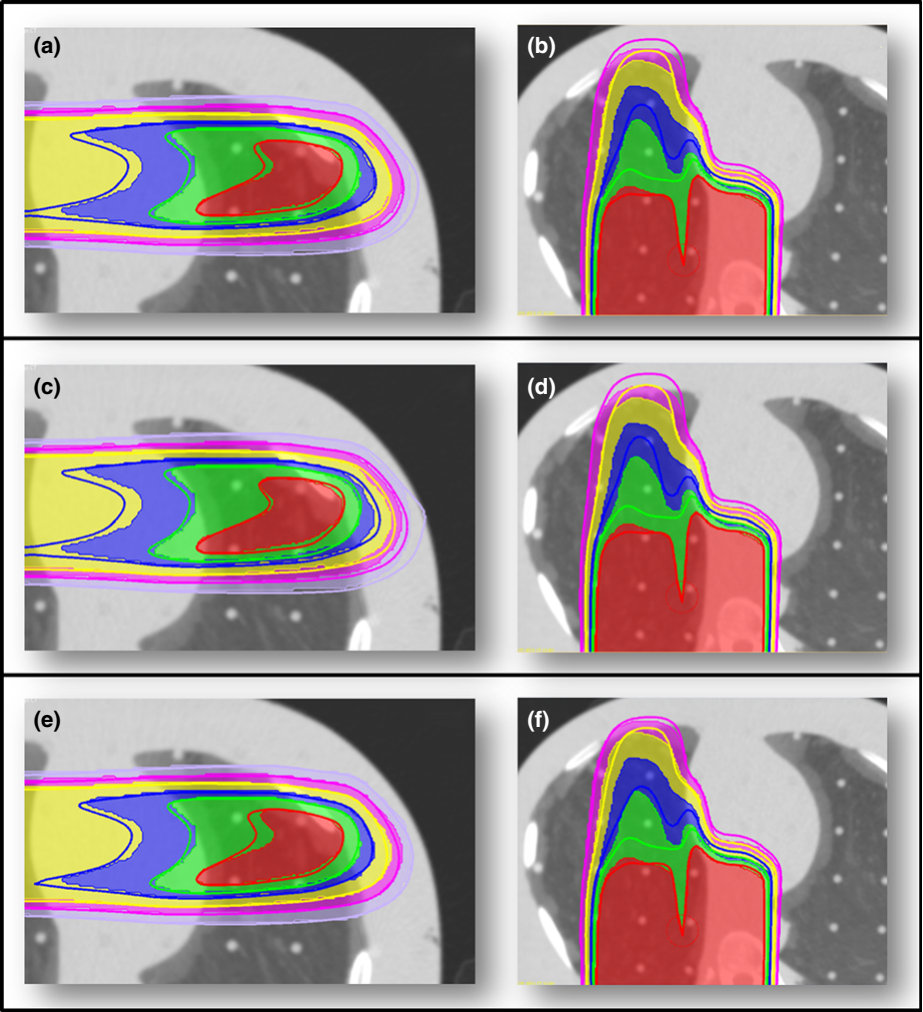
AIRO verification plans for RANDO Thorax AP and Lat plans: (a‐b) CT Sim SPR curve, (c‐d) AIRO Stoichiometric curve, and (e‐f) dose‐adjusted curve. The isodose with thin line represents the dose from the AIRO CT scan. The thick line represents the isodose lines from the original treatment planning CT. The red, green, blue, yellow, purple, and lavender lines represent: 20, 18, 16, 12, 8, and 4 Gy, respectively.

Initial visual inspection was performed to evaluate the dose comparison between AIRO CT calculation and initial CT Sim dose calculations. Special attention was focused on the end of range for the single beam plans. Dose calculated on the RANDO lung phantom with a single lateral field is illustrated in Fig. 4. The treatment planning isodose lines were converted to contours and are illustrated with the thick lines. Dose calculated with the clinically commissioned SPR curve for the CT Sim is illustrated compared to treatment planning dose in Fig. 4(a). Note that the dose on the AIRO CT extends deeper by about 5 mm compared to the original treatment plan. Dose was then calculated using the Stoichiometric AIRO SPR curve [Fig. 4(c)]. Based on data reported in Fig. 3 indicating changes in the CT number for low‐density plugs depending on the configuration of the phantom, these values were changed until the end of range had better comparison between the treatment planning CT and the AIRO CT dose calculations. The final result of the SPR curve is shown in Fig. 3 and the dosimetric result is illustrated in Fig. 4(e). Figures 4(b), 4(d), and 4(f) show similar trend for a field placed posteriorly on the RANDO phantom.

#### 3.B.2 Water‐equivalent thickness comparisons

Water‐equivalent thickness (WET) (gm/cm^2^) values along the PA lung beam on the RANDO thorax phantom demonstrated results shown in Table 3 for the CT Sim, Stoichiometric AIRO, and dose‐adjusted SPR curves. WET for the beam on the pelvis RANDO phantom demonstrated results shown in Table 4 for the CT Sim, Stoichiometric AIRO, and dose‐adjusted SPR curves. Average percent differences for the RANDO pelvis phantom were as follows: 0.32 ± 1.49% (CT Simulator vs. AIRO with CT Simulator SPR curve), 0.44 ± 1.30% (CT Simulator vs. AIRO with Stoichiometric AIRO SPR curve), and 0.73 ± 1.36% (CT Simulator vs. AIRO with dose‐adjusted curve). Average percent differences for the RANDO thorax phantom were as follows: 1.61 ± 2.80% (CT Simulator vs. AIRO with CT Simulator SPR curve), 2.48 ± 2.35% (CT Simulator vs. AIRO with Stoichiometric AIRO SPR curve), and −0.28 ± 3.85% (CT Simulator vs. AIRO with dose‐adjusted curve).

**Table 3:** WET values in cm for PA beam on RANDO Thorax phantom

CT Sim scan	AIRO scan		
	CT Sim curve	AIRO curve	Dose curve
1.13	1.07	1.07	1.07
3.43	3.35	3.29	3.37
2.89	2.68	2.69	2.77
5.64	5.63	5.64	5.54
4.90	4.88	4.78	5.11
9.82	9.75	9.72	9.74
6.69	6.74	6.60	6.99
16.95	16.93	16.90	16.89
7.94	7.91	7.80	8.30
9.93	10.00	9.84	10.37

**Table 4:** WET values in cm for beam on RANDO Pelvis phantom

CT Sim scan	AIRO scan		
	CT Sim curve	AIRO curve	Dose curve
0.84	0.84	0.84	0.82
3.10	3.01	3.00	3.00
5.14	5.07	5.05	5.05
7.27	7.44	7.34	7.32
9.75	9.76	9.76	9.74
11.91	11.87	11.85	11.84
13.95	14.08	14.07	14.05
15.03	15.10	15.08	15.06
17.07	16.80	17.03	17.02
25.00	25.03	24.99	24.95

#### 3.B.3 Nonclinical plans

The gamma analysis comparing the CT Sim treatment plan to each of the AIRO calculated plans for different gamma thresholds was averaged over the three planes passing through the beam isocenter (coronal, sagittal, and transverse). This was done to eliminate bias in the beam direction so the different plans on different phantoms could be compared. Data were also averaged between two AIRO CT scans for each phantom. A total of six different single beam plans were evaluated. Average percentage of passing points based on Gamma criteria of 1%,/1 mm, 2%/2 mm, and 3%,/3 mm are shown in Table 5 for the six different cases. Gamma analysis between the treatment plans and AIRO verification plans for single beam delivery showed that the dose‐adjusted SPR curve was slightly better than the Stoichiometric AIRO curve; however, using the original treatment planning curve also yields good agreement. The relatively high passing rate for the dose‐adjusted method indicates that calculated proton dose on AIRO image sets is sensitive enough to monitor dosimetric changes that warrant plan adaptation (Table 5).

**Table 5:** Gamma analysis percent of passing points comparing nonclinical dose for treatment plans developed on (1) CT Sim vs. AIRO with clinical CT Sim SPR curve, (2) AIRO SPR curve, and (3) dose‐adjusted curve

	1%, 1 mm (%)			2%, 2 mm (%)			3%, 3 mm (%)		
	CT Sim	AIRO	Dose	CT Sim	AIRO	Dose	CT Sim	AIRO	Dose
RANDO Pelvis	98.5	97.8	96.6	99.9	99.8	99.7	100.0	100.0	100.0
RANDO PA Thorax	77.9	77.3	79.0	86.1	87.1	87.4	91.0	90.1	91.7
RANDO Lat Thorax	71.1	60.3	83.1	99.0	98.6	98.3	99.6	97.3	99.4
RANDO Head	96.2	95.3	95.2	99.5	99.5	99.5	99.6	99.6	99.6
CIRS Lung	92.8	92.1	93.1	97.7	97.5	98.6	98.8	98.5	99.6
CIRS Head	77.7	77.1	77.0	92.9	92.6	92.7	97.1	96.7	96.8

At 3%/3 mm, the dose‐adjusted SPR curve provided equivalent or higher passing points than the CT Sim and the stoichiometric curves for the RANDO pelvis & Head fields, the RANDO PA Lung field, and the CIRS Lung phantom. For the remaining two phantoms (RANDO Lat Thorax, and CIRS Head) where the dose‐adjusted curve did not provide the highest or equivalent number of passing points, the difference between the dose‐adjusted curve and CT Sim or stoichiometric SPR curve's passing points was ≤0.3%. At 1%/1 mm, the dose‐adjusted SPR curve provided equivalent or higher passing points than the CT Sim and stoichiometric SPR curves for the RANDO Lat Thorax (83.1% passing points vs. 71.1% passing points), RANDO PA Thorax (79.0% passing points vs. 77.9% passing points), and CIRS Lung (93.1% passing points vs. 92.8% passing points). For the remaining three phantoms where the dose‐adjusted curve did not provide the highest or equivalent number of passing points (CIRS Head, Rando Pelvis, and RANDO Head), the difference between the dose‐adjusted curve and CT Sim or Stoichiometric AIRO SPR curve's passing points was ≤1.9%. In each of these cases, the CT Simulator SPR Curve provided the highest number of passing points.

#### 3.B.4 Clinical plans

**Figure 5:**
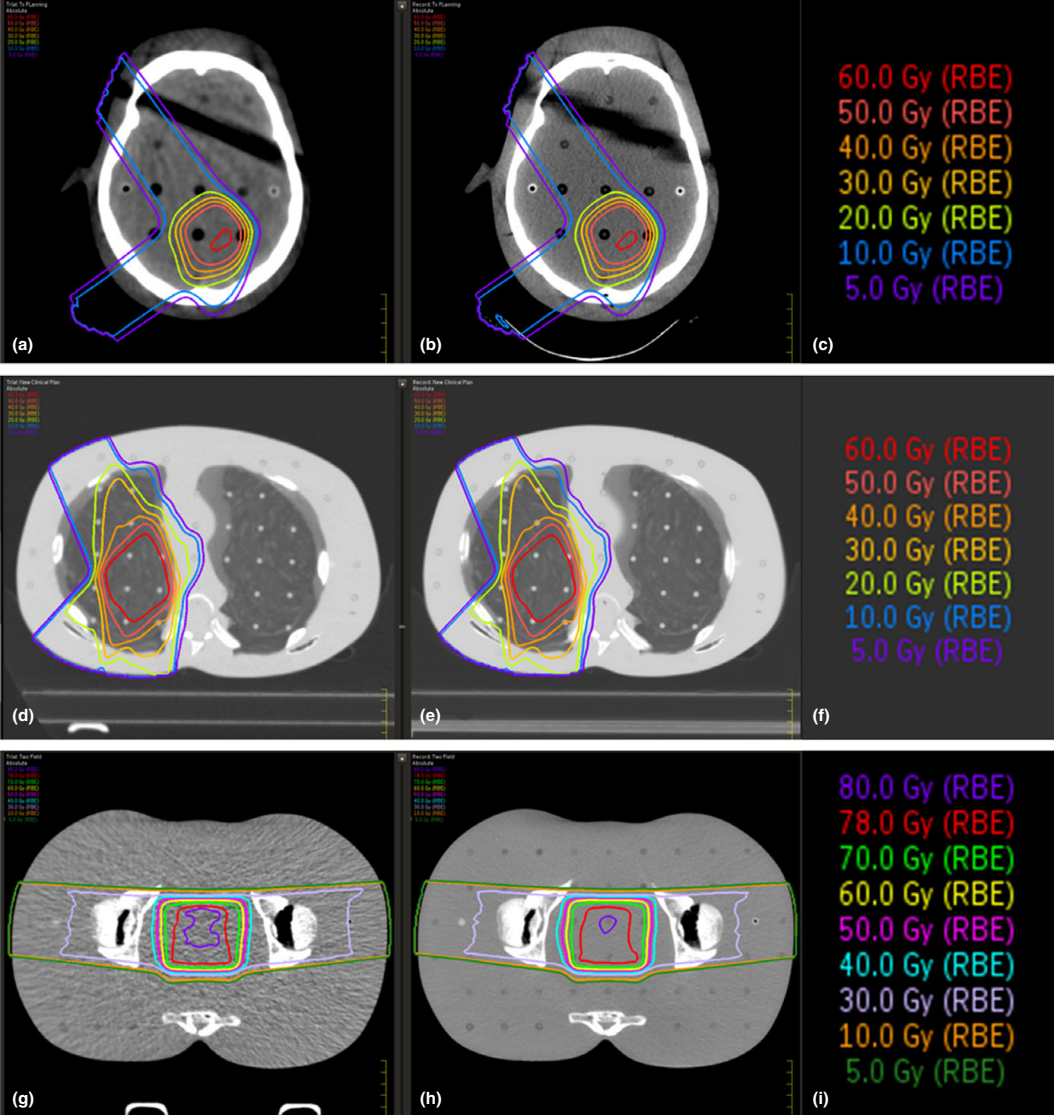
Isodose lines for clinical treatment plans in the RANDO head (a,b,c), thorax (d,e,f), and pelvis phantoms (g,h,i) for the AIRO images with the dose‐adjusted SPR curve (left) (a,d,g) and the CT Sim images with CT Sim SPR curve (right) (b,e,h).

The clinical treatment plans are shown in Fig. 5 and compare the AIRO adapted treatment plan on the left compared to the treatment planning CT plan on the right. Gamma analysis between clinical treatment plans and AIRO verification plans demonstrated that the dose‐adjusted method provided better dose agreement at 1%/1 mm for the RANDO head plan. The CT Sim SPR curve provided superior dose agreement at 1%/1 mm for the RANDO pelvis, RANDO thorax, and CIRS lung plans. The CIRS Lung phantom exhibited the largest passing point differences of 6.2% between the dose‐adjusted curve and the CT Sim SPR curve. However, at 3%/3 mm the CIRS Lung phantom demonstrates equivalent dose to the CT Sim and AIRO Stoichiometric SPR curve. The Stoichiometric AIRO curve demonstrated slightly higher target coverage than the dose mapped curve with identical mean and maximum dose (58.9 ± 1.2 Gy). The AIRO‐specific stoichiometric SPR curve provided superior dose agreement for the CIRS head phantom at 1%, 1 mm. Insignificant dose differences were found at 2%/2 mm or 3%/3 mm criteria for RANDO pelvis or RANDO head. At 3% and 3 mm, there were no difference in points passing in the CIRS lung or CIRS head phantom. For a criteria of 3% and 3 mm, any of the SPR curves are sufficient (Table 6). However, we recommend that clinicians investigate the CT number variability in the low‐density region and investigate the dosimetry in this region. Of note for the clinical plans, dose–volume histograms (DVHs) for structures of interest near the target volume showed no change from one SPR curve to another.

**Table 6:** Gamma analysis percent of passing points comparing clinical dose for treatment plans developed on (1) CT Sim vs. AIRO with clinical CT Sim SPR curve, (2) AIRO SPR curve, and (3) dose‐adjusted curve

	1%, 1 mm (%)			2%, 2 mm (%)			3%, 3 mm (%)		
	CT Sim	AIRO	Dose	CT Sim	AIRO	Dose	CT Sim	AIRO	Dose
RANDO Pelvis	95.6	95.5	95.0	100.0	100.0	100.0	100.0	100.0	100.0
RANDO Thorax	85.9	75.8	81.7	97.6	96.0	95.8	99.1	98.9	98.6
RANDO Head	99.5	99.2	99.7	99.9	99.9	99.9	99.9	99.9	99.9
CIRS Lung	88.8	81.0	82.6	99.9	98.0	98.6	99.8	99.8	99.8
CIRS Head	90.3	91.5	91.0	98.6	98.7	98.6	100.0	100.0	99.5

## 4 DISCUSSION

This study shows that proton dose calculations on CT images sets from the AIRO mobile CT system can be used to calculate dose with relatively high accuracy similar to the clinically commissioned CT Sim. Therefore, in principle, the system can be used for adaptive proton treatment planning. Prior to this study, we developed many preliminary tests to determine which factors most affected the AIRO's CT numbers.^3^ We tested various plug configurations, mA, table positions, reconstruction kernels, FOV, and phantoms. We found that reconstruction artifacts were minimal and that CT numbers were mostly affected by the surrounding materials and plug patterns. Thus, we decided to use the plug pattern suggested by Gammex when building the AIRO's SPR curve and we tested the AIRO CT numbers with various surrounding materials.

We found that AIRO HU values of both small lung plug volumes changed up to 6.3% (LN 450) when surrounded by solid water—which was not reproduced with our CT Sim (up to 1.7% difference). The reason for this phenomenon with AIRO scans is still unclear but could be related to the reconstruction of the images and beam hardening. This could also be related to the fact that the AIRO has bigger bore compared to a conventional CT simulator which introduces more scatter issues. The magnitude of these differences in air‐like media is quantified.^3^ Therefore, we believe that dosimetric equivalency testing using visual display of isodose lines and WET values between the planning CT scanner and in‐room CT scanner is necessary for all in‐room CT‐based imaging systems before they are deployed for adaptive planning purposes. Investigation of the WET for beams traversing through longer paths of heterogeneity indicated that slight adjustments to the SPR curve could help to improve the coincidence of the distal fall‐off of the curve. Especially in the situation for longer path length and beams stopping in low‐density material where the CT values for the AIRO were not consistent with the CT values of our Philips scanner. In our situation, changes had to be made in the low‐density area of the stopping power curve to get the dosimetric curves and the WET to match more closely for beams passing through lung. Differences were also found in the cortical bone CT values, but this did not appear to affect the WET for the pelvis phantom and therefore the SPR curve was only adjusted slightly for the low‐density region. Also, more attention was paid to the low‐density area of the curve as the focus for dynamic planning is in clinical cases involving lung and tumors in the lung where tumor breakdown and/or fluid buildup would dramatically change the WET of the proton beam.

Proton beam dose distribution in tissue is extremely sensitive to variations in path length due to either real changes in amount of material in the beam path (patient anatomical changes) or variations in tissue HU values due to CT scanner reconstruction and acquisitions variations, or both. In order to quantitatively assess the effect of both variables on proton dose distributions, the use of a combined dose and range criteria is necessary. Differences in HU values for the same phantom using two different CT scanners are known to cause a range uncertainty in the 1%–3% range.^11^, ^12^ Dose, range, and stopping power are strongly correlated in proton beams. A change in the order of 1%–3% in stopping power ratio due to HU changes can translate to 1%–3% dose difference in the middle of SOBP and (ICRU 78).^17^ For a clinical beam on the order 10 cm range, 1%–3% a change in beam range (1–3 mm) can translate to a dose difference at the distal end of the beam by as much as 30% due to the sharp distal penumbra. Therefore, the use of 1%–3% dose difference and 1–3 mm range difference comparison criteria is appropriate and a sensitive indicator to correlate differences in dose for the same plan calculated on different CT data sets. Dose discrepancies at the end of range specifically for beams traversing through low‐density tissue that were used as the bases to adjust the SPR curve to create the dose‐adjusted SPR curve so that the agreement would be within 1–2 mm in the high‐gradient region where the dose difference would be great. The failing points in the gamma analysis are mostly in the low‐gradient region where the dose is different by >3% due to image noise and changes in the low‐density region.

Our results demonstrated that performing the stoichiometric analysis for a given phantom and CT scan may not provide dose equivalence between two different CT scans. For the purposes of using an in‐room CT for adaptive planning, it was important to verify the dosimetric equivalence of the two CT image sets with their corresponding stopping power curves. This was only achieved by directly mapping CT values and subsequently adjusting them to yield better dosimetric comparisons at the end of range. For robust proton beams (e.g., multiple beams, through less heterogeneity) with appropriate range uncertainty margins, the overall difference between the two CT images becomes less important.

At the time of writing this manuscript, the AIRO has been utilized for localization and/or planning QA on several lung, head and neck, and breast patients. The AIRO workflow adds approximately 5–10 min to the daily treatment when used for localization and provides the added benefit of aligning the patient in all six degrees‐of‐freedom. In addition to using the CT scan for image alignment and verification scanning, the AIRO CT scans have been used for offline dose‐adaptation in some cases where the physician deems in necessary due to clinical changes in anatomy. This can be done by changing the range and modulation in cases where the WET changes by several mm and also be evaluating coverage on the dose–volume histograms. With the AIRO imaging capability, the patient would not have any delay in starting a re‐plan. This work lays the groundwork for true CT‐based adaptive planning for any proton therapy system.

## CONCLUSIONS

We present a methodology for developing a stopping power calibration curve on a new in‐room AIRO mobile CT scanner for the purpose of adaptive proton therapy. Our methodology is based on re‐iterative dose‐based mapping of SPR values between the clinically commissioned stationary CT scanner and AIRO for a variety of phantom, This approach yielded better overall dosimetric equivalency to the conventional stoichiometric method based on dose calculations in various heterogeneous phantoms. We show that the AIRO CT system can be a viable alternative to conventional CT Sim for the purpose of adaptive planning in proton therapy.

## CONFLICT OF INTEREST

The authors declare no conflict of interest.

## References

[1] Szeto Y , Witte M , van Kranen S , Sonke J , Belderbos J , van Herk M . Effects of anatomical changes on pencil beam scanning proton plans in locally advanced NSCLC patients. Radiother Oncol. 2016;120:286–292. 27393217 10.1016/j.radonc.2016.04.002

[2] Kurz C , Nijhuis R , Reiner M , et al. Feasibility of automated proton therapy plan adaptation for head and neck tumors using cone beam CT images. Radiat Oncol. 2016;11:1–9. 27129305 10.1186/s13014-016-0641-7 PMC4851791

[3] Oliver JA , Zeidan O , Meeks SL , et al. The Mobius AIRO Mobile CT for image‐guided proton therapy: characterization & commissioning. J Appl Clin Med Phys. 2017;18:1–7. 10.1002/acm2.12084 PMC5689854 28436155

[4] Weir VJ , Zhang J , Bruner AP . Dosimetric characterization and image quality evaluation of the AIRO mobile CT scanner. J X‐Ray Sci Technol. 2015;23:373–381. 10.3233/XST-150496 26410470

[5] Veiga C , Janssens G , Teng C , et al. First clinical investigation of cone beam computed tomography and deformable registration for adaptive proton therapy for lung cancer. Int J Radiat Oncol Biol Phys. 2016;95:549–559. 27084664 10.1016/j.ijrobp.2016.01.055

[6] Landry G , Dedes G , Zöllner C , et al. Phantom based evaluation of CT to CBCT image registration for proton therapy dose recalculation. Phys Med Biol. 2014;60:595. 25548912 10.1088/0031-9155/60/2/595

[7] Veiga C , Janssens G , Baudier T , et al. A comprehensive evaluation of the accuracy of CBCT and deformable registration based dose calculation in lung proton therapy. Biomed Phys Eng Express. 2017;3:015003.

[8] Jin X , Hu W , Shang H , et al. CBCT‐based volumetric and dosimetric variation evaluation of volumetric modulated arc radiotherapy in the treatment of nasopharyngeal cancer patients. Radiat Oncol. 2013;8:1–7. 24289312 10.1186/1748-717X-8-279 PMC4222038

[9] Kim J , Park Y‐K , Sharp G , Busse P , Winey B . Water equivalent path length calculations using scatter‐corrected head and neck CBCT images to evaluate patients for adaptive proton therapy. Phys Med Biol. 2016;62:59–72. 27973351 10.1088/1361-6560/62/1/59 PMC5397286

[10] Park Y‐K , Sharp GC , Phillips J , Winey BA . Proton dose calculation on scatter‐corrected CBCT image: feasibility study for adaptive proton therapy. Med Phys. 2015;42:4449–4459. 26233175 10.1118/1.4923179 PMC4517932

[11] Moyers M , Miller D , Bush D , Slater J . Methodologies and tools for proton beam design for lung tumors. Int J Radiat Oncol Biol Phys. 2001;49:1429–1438. 11286851 10.1016/s0360-3016(00)01555-8

[12] Paganetti H , Athar BS , Moteabbed M , Adams JA , Schneider U , Yock TI . Assessment of radiation‐induced second cancer risks in proton therapy and IMRT for organs inside the primary radiation field. Phys Med Biol. 2012;57:6047–6061. 22968191 10.1088/0031-9155/57/19/6047

[13] Low DA , Dempsey JF . Evaluation of the gamma dose distribution comparison method. Med Phys. 2003;30:2455–2464. 14528967 10.1118/1.1598711

[14] Song JH , Shin H‐J , kay CS , Chae S‐M , Son SH . Comparison of dose calculations between pencil‐beam and Monte Carlo algorithms of the iPlan RT in arc therapy using a homogenous phantom with 3DVH software. Radiat Oncol. 2013;8:1–11. 24305109 10.1186/1748-717X-8-284 PMC4235017

[15] Ojala JJ , Kapanen MK , Hyödynmaa SJ , Wigren TK , Pitkänen MA . Performance of dose calculation algorithms from three generations in lung SBRT: comparison with full Monte Carlo‐based dose distributions. J Appl Clin Med Phys. 2014;15:4662. 24710454 10.1120/jacmp.v15i2.4662 PMC5875463

[16] Chung JB , Kim JS , Ha SW , Ye S‐J . Statistical analysis of IMRT dosimetry quality assurance measurements for local delivery guideline. Radiat Oncol. 2011;6:1–8. 21439096 10.1186/1748-717X-6-27 PMC3073875

[17] ICRU Report 78 Prescribing, Recording, and Reporting Proton‐Beam Therapy International Commission on Radiation Units and Measurements; 2007.

